# Leading the Charge on Digital Regulation: The More, the Better, or Policy Bubble?

**DOI:** 10.1007/s44206-023-00033-7

**Published:** 2023-01-17

**Authors:** Cristiano Codagnone, Linda Weigl

**Affiliations:** 1 Department of Social and Political Sciences, Università Degli Studi Di Milano, Milan, Italy; 2 Interdisciplinary Centre for Security, Reliability and Trust, University of Luxembourg, Luxembourg, Luxembourg

**Keywords:** Digital policy, Digital sovereignty, Digital constitutionalism, Policy bubbles

## Abstract

For about a decade, the concept of ‘digital sovereignty’ has been prominent in the European policy discourse. In the quest for digital sovereignty, the European Union has adopted a constitutional approach to protect fundamental rights and democratic values, and to ensure fair and competitive digital markets. Thus, ‘digital constitutionalism’ emerged as a twin discourse. A corollary of these discourses is a third phenomenon resulting from a regulatory externalisation of European law beyond the bloc’s borders, the so-called ‘Brussels Effect’. The dynamics arising from Europe’s digital policy and regulatory activism imply increasing legal complexities. This paper argues that this phenomenon in policy-making is a case of a positive ‘policy bubble’ characterised by an oversupply of policies and legislative acts. The phenomenon can be explained by the amplification of values in the framing of digital policy issues. To unpack the policy frames and values at stake, this paper provides an overview of the digital policy landscape, followed by a critical assessment to showcase the practical implications of positive policy bubbles.

## Introduction

Shortly after reports of surveillance by foreign governments spread in 2013, many European countries issued legislative proposals or commissioned policy reports that fall under a broadly defined concept of *digital sovereignty* (Federal Ministry for Economic Affairs and Energy, 2021; Federal Ministry for Digital and Economic Affairs, 2020; Merkel et al., 2021). Already in 2014, dozens of such proposals emerging in countries such as Germany and France were mapped (Maurer et al., 2014). They ranged from establishing undersea cables, and national e-mail systems, to localised data storage, localised routing, and the like.

Unpleasent scandals and news, such as Cambridge Analytica or the 2013 revelations about some European political leaders having been wiretapped, acted as catalysts for the emergence of European discourses on technological and digital sovereignty. Previously vaunted features of digital connectivity, such as anonymity, openness, and centralization, soon evolved into threats that prompted countries to act to preserve their digital state sovereignty. In 2017, in his Sorbonne speech, Emmanuel Macron referred to digital technology as the ‘fifth key’ to Europe’s sovereignty. “Rather than bemoaning the fact that the current leaders in the digital technology are American, to be followed by the Chinese”, he argued, “we must create European champions” (Macron, 2017). The concept of digital sovereignty has ever since figured prominently in the European policy discourse (Couture & Toupin, 2019; Möllers, 2020; Mueller, 2020; Pohle & Thiel, 2020; Timmers, 2021). Digital sovereignty or *digital autonomy* has subsequently become mainstreamed in both speeches and policy documents of the European Commission.^1^ In her November 2019 inauguration speech, Commission President von der Leyen announced *digital* as one of the European Union’s (EU) top priorities for the next 5 years. According to her guidelines, Europe needs to have mastery and autonomy in key technological areas, such as quantum computing, artificial intelligence (AI), blockchain, or critical chip technologies. In March 2021, the Commission unveiled its vision for a human-centred, prosperous, and sovereign digital future by 2030: the Digital Compass. An example of this discourse is the presentation of Europe’s goal to build a fully self-sufficient cloud computing infrastructure through the Gaia-X project to ensure European data sovereignty (Federal Ministry for Economic Affairs and Energy, 2019). As a federated data infrastructure, the Gaia-X ecoystem also offers opportunities for the AI market. The EU has mused about the creation of an ‘Airbus for AI’ (Pohl, 2021) presumably as a historical reminiscence of the European Airbus that challenged US manufacturers in the 1960s. By connecting small and medium-sized cloud providers in Europe through a common standard, the AI project would ostensibly offer an open, secure and trusted European alternative to the world’s largest (often US-based) cloud service providers (e.g., Amazon, Google, Microsoft), while at the same time respecting European values and data protection standards. Another example is the legislative proposal to establish a framework for a European Digital Identity presented by the Commission in June 2021. The proposed pan-European digital identity management system forms another element of Europe’s strategy to counter the hegemonic positions of non-European multinational technology companies (Weigl et al., 2022). With the increasing number of legislative proposals, a twin discourse to digital sovereignty at EU level, that of *digital constitutionalism*, emerged (Celeste, 2019; De Gregorio, 2021). As follow-ups to the General Data Protection Regulation (GDPR), the AI Act, the Digital Services Act (DSA), the Digital Markets Act (DMA), the Data Governance Act (DGA), and the Data Act (DA) form the extraterritorial pillars (Scott, 2014) of a new EU digital constitutionalism. Under this approach, legal acts are no longer limited to or applicable within European territory, but affect any activity involving EU citizens or businesses. In transcending traditional territoriality, such approach has been deemed post-Westphalian (Floridi, 2014). A corollary of these discourses is one that emerges as the so-called *Brussels Effect* (Bradford, 2020). The Brussels Effect implies that market participants must comply with EU regulations regardless of where they operate if their activities affect EU citizens. If they do not want to forego a market of 500 million consumers, companies will have to adopt the requirements of the applicable EU regulations. As such, the Brussels Effect consists in influencing the global digital domain primarily through market mechanisms (Damro, 2012) and credible regulatory capacity (Cervi, 2022).

Against this background, we have witnessed strong European activism in the development of both policies and legislative acts over the past 5 years (Pagallo, 2022). In the domain of AI, for instance, the Commission actively produced several policy documents and communications (Jacobs & Simon, 2022) that culminated in the AI Act. The European Data Protection Supervisor defined the AI Act as “the first initiative, worldwide, that provides a legal framework for Artificial Intelligence” and considered it to be “one of the most influential regulatory steps taken so far internationally” (Floridi, 2021, p. 216). The EU arsenal of digital policies and digital legislative acts is frequently legitimised within the discourse of digital sovereignty. However, the scholarly debate has observed that Europe’s “regulatory global influence [constitutes] by no means [a] sufficient step toward attaining and maintaining digital sovereignty” (Metakides, 2022, p. 222) and that the narratives on sovereignty should be taken with caution (Floridi, 2020; Metakides, 2022; Pagallo, 2022).

In this paper, we argue that the EU’s *regulatory activism* in digital policy-making is a case of a *positive policy bubble* characterised by an *oversupply* of policies and legal acts, as opposed to policy *underreaction* driven by emotional sentiments in the case of a *negative policy bubble*. The positive policy bubble is to be explained by the amplification of positive, supporting values in the framing of the policy issues at stake. We therefore analyse the policy bubble and key framing mechanisms within the discourse on digital sovereignty and digital constitutionalism. We also argue that such digital policy and regulation activism is becoming increasingly complex, making regulatory coherence and consistency difficult to achieve. In doing so, this paper first reviews the digital policy landscape around European legislation and its associated frames and values. It then provides a critical assessment of digital policy oversupply. The paper concludes by presenting the socioeconomic and legal implications of positive policy bubbles in the EU.

## Digital Sovereignty and Digital Constitutionalism in the European Union

The emergence of sovereignty in the digital sphere was introduced more than three decades ago. *Cyber-sovereignty* exhibits how the emergence of technology and networks affect the international order and challenges the perception of Westphalian sovereignty as a ubiquitous principle from two main perspectives (Pohle & Thiel, 2020; Mueller, 2020). The first challenge, *cyber exceptionalism*, presumes that the profileration of technologies and the solidification of cyber space implies the demise of state sovereignty and governmental interference (Pohle & Thiel, 2020). The defenders of cyber exceptionalism express distrust towards established political institutions and argue that networks would better meet the requirements of modern societies than traditionally centralised forms of organization. The second challenge, *multi-stakeholder internet govern*ance, focuses on the non-sovereign roles that states play in a regulatory ideal that corresponds to the diffuse and transnational constellations of global digital networks, such as bottom-up collaboration and consensual decision-making (Pohle & Thiel, 2020).

Today, with digital technology sweeping into every aspect of society, we witness a resurgence of sovereignty as a principle of digital policy-making. Concerns about whether governments exercise too much power and control on the Internet are fading (Maurer et al., 2014; Thiel, 2014). Instead, questions about Internet governance (Deibert, 2009), geopolitics, and cybersecurity (Ebert & Maurer, 2013) replaced the debate to a large extent. Scholars investigate competing efforts by states to pioneer cyberspace from the perspective of various motivations, including concerns over national security, political pressures, or the protection of cultural, social and economic values (Deibert, 2009; Ebert & Maurer, 2013; Maurer et al., 2014; Floridi, 2020).

With the global surveillance disclosures, notably the leaks by whistleblower Edward Snowden, as well as the growing influence of social media platforms and e-commerce businesses, the ungovernability of cyberspace became a widely perceived issue for regulatory regimes. The anonymity, openness, and centralization of the Internet turned into a strategic problem that threatened to result in surveillance, wars, terrorism, and crime, rendering digital state sovereignty obligatory. In light of these trends, sovereignty backed by the idea of a state’s authority over a demarcated territory became an anachronistic concept, and the contemporary meaning of digital sovereignty embodied a new purpose (Mueller, 2020; Pistor, 2020; Pohle & Thiel, 2020). This new purpose ought to address rising user demands, as well as increasing security risks and privacy violations by governments and multinational technology corporations. Hence, scholars began to focus on individuals’ digital rights, data protection and economic competitiveness, thus tailoring the contextualization of sovereignty towards the microlevel (Metakides, 2022; Pinto, 2018; Couture & Toupin, 2019; Pohle & Thiel, 2020; Floridi, 2020). In addition to that, many nation states felt challenged by a digital transformation that was disproportionally driven by non-European, gigantic private technology companies (Ciaran, 2022; Floridi, 2020; Pistor, 2020). This created a dependence of public institutions on the services and products delivered private companies, positioning the EU in the geopolitics of technology as a ‘non-aggressive’, stabilising, regulatory power (Ciaran, 2022; Metakides, 2022) with a self-imposed mandate to achieve digital sovereignty. However, it is necessary to acknowledge a certain degree of ambiguity and inadequacy of digital sovereignty as a legal reference in the context of supranational EU policy-making (Pagallo, 2022). Particularly because in an effort to attain digital sovereignty, the EU opted for a constitutional approach to protect fundamental rights and democratic values (De Gregorio, 2021; Floridi, 2021).

Digital constitutionalism is used to explain the emergence of regulatory counteractions against the technological and digital challenges of our time. From a legal perspective, technology not only amplifies and threatens existing rights and obligations, but also triggers the need and recognition of new ones (Celeste, 2019). This is being responded to by normative counteractions in an effort to restore “a condition of relative equilibrium in the constitutional ecosystem” (ibid., p. 5). As examples of such counteractions, Celeste (2019) lists the right to Internet access, the emergence of data protection laws, transparency obligations and the right to audit institutions and access public information. However, the concept of digital constitutionalism is not new, and Celeste (2019) recognises a lack of consensus within the previous literature on digital constitutionalism. Some scholars interpret the concept as a limitation of private power (Berman, 2000; Fitzgerald, 1999; Suzor, 2018), others as a limitation of the power of public authorities (Gill et al., 2015). Moreover, it is unclear whether digital constitutionalism is a concept restricted to private law, constitutional law, or a hybrid thereof (Padovani & Mauro, 2018). According to Celeste, the concept of digital constitutionalism embodies an ideology that seeks to tailor the values of contemporary constitutionalism to the disruptive impact of digital technology by limiting the power of both public and private actors who emerge as increasingly dominant players (2019). He highlights that “the notion of digital constitutionalism should not be used to term concrete normative instruments” and “does not exclusively involve a formal institutionalisation or codification of norms in binding legal texts” (p. 18). Instead, as a new strand of contemporary constitutionalism, it discusses and elaborates on foundational values and constitutional principles in the context of digital technology.

Floridi adds that with the growing number of legislations in the digital realm, digital constitutionalism is especially felt in Europe (2021) with regulatory influence exerted not only within but also outisde its boundaries (Cervi, 2022). De Gregorio (2021) explains the reasons and paths behind the constitution-oriented approach in the EU through a three-step chronology of digital liberalism, judicial activism, and digital constitutionalism. He argues that digital constitutionalism, as a result of the previous two phases, is a response to constitutional threats posed by powerful transnational corporations operating in the digital environment. As such, EU digital constitutionalism is based on the codification of the European Court of Justice’s mission to protect fundamental rights and democratic values, and on the effort to limit online platforms’ powers through regulatory instruments (De Gregorio, 2021; Floridi, 2021). However, the increasing number of policies regulating digital is not only due to institutional metamorphosis, as the example of the Court’s emerging role as a human rights adjudicator indicates. Policy-making processes play a fundamental role, as our conceptual framework in the following section will show.

## Conceptual Framework: Policy Bubbles, Frames, and Values

The pursuit of Europe’s digital sovereignty, the discourses of digital constitutionalism, and the legacies of the Brussels Effect have contributed to digital policy-making activism with complex legal consequences (Celeste, 2019; De Gregorio, 2021). These developments are affecting regulatory coherence and consistency, an area where the EU specifically appears to struggle (Brownsword, 2019). We interpret this occurrence using the concept of policy bubbles in combination with that of policy framing (Jones et al., 2014; Maor, 2014, 2016). In this context, the EU digital policy and legislation activism represent a case of a positive policy bubble with an oversupply of policy initiatives and legislative acts, explained by the amplification of values in the framing of the issues at stake.

Maor elaborates on both negative and positive policy bubbles. The notion of negative policy bubbles explains the systematic undersupply of policies due to negative emotional sentiments in public policy processes (Maor, 2016). A positive policy bubble occurs when policies become valued for reasons not strictly related to the possibility to achieve policy goals, but rather for symbolic or ideological reasons (Jones et al., 2014, p. 149). In a way, politics and values prevail over traditional reasoning, and framing contributes to creating the cognitive and affective bases for the bubble to take hold (Maor, 2014, 2016). The emergence of a positive policy bubble typically follows one of these dynamics: (1) an endogenous process that affects opinion formation, attention, learning, behaviour, and attitudes; (2) an exogenous shock that triggers an endogenous process; (3) the adoption of policy framing, or (4) a process by which the framing context within which the policy process takes place conditions policy dynamics.

The framing perspective, first adopted in studies of communication and rhetoric (Entman, 1991; Kyupers, 2009) and in the study of social movement (Benford & Snow, 2000; Klandermans, 1998; Snow et al., 1986), has been more recently applied to policy-making (Béland, 2009; Béland & Cox, 2016), including in a research program on policy framing in the EU and the US (Baumgartner & Jones, 2009; Baumgartner & Mahoney, 2008; Chong & Druckman, 2007; Daviter, 2007). Since policy issues are multidimensional, different readings and conceptualisations are possible, making them malleable along various perspectives. Thus, influencing policies is important (Chong & Druckman, 2007, p. 104). If one policy frame wins over alternative ones in the course of the policy-making process, this will influence and shape the initiatives that will be brought forward. In particular, *frame alignment*, *frame bridging*, and *frame amplification* are processes of strategic importance (Snow et al., 1986). Frame alignment occurs when individual frames are linked and thus produce *frame resonance* to catalyse consensus. Frame bridging involves the “linkage of two or more ideologically congruent but structurally unconnected frames regarding a particular issue” (Snow et al., 1986, p. 467). Frame amplification refers to “idealization, embellishment, clarification, or invigoration of existing values or beliefs” (Benford & Snow, 2000, p. 624). Beliefs are convictions or assumptions people make about the world. They provide the context from which important and stable societal principles emerge, which eventually manifest themselves as values. Therefore, values are fairly pervasive and integrated standards into society. They have “considerable staying power” (p. 613), and provide a reasonable and well-grounded frame for positive policy bubbles.

Yet, as suggested by Snow et al. (1986), actors involved in framing processes can do more than just refer to a value or a belief central to a society’s cultural repertoire. Value amplification refers to the identification, idealisation, and elevation of one or more values presumed to be basic to prospective constituents. Surel (2000) has critically appraised the trend that emphasises the influence of cognitive and normative elements in public policy-making. While acknowledging that such ideational and framing perspectives encounter some institutional constraints, Surel recognises their importance in integrating previously separated normative and cognitive analysis (2000). According to Béland (2009), framing affects the policy-making process in three ways: (a) by constructing the issues entering the agenda; (b) by shaping the assumptions that affect the content of policy proposals; and (c) by building discursive weapons in the construction of reform imperatives.

We argue that in what we consider the EU positive policy bubble, the key framing mechanism has been value amplification applied to the discourse of digital sovereignty. This became subsequently linked to those of digital constitutionalism and of the Brussels Effect.

## Digital Policy Activism in Brussels: a Selective Review and Assessment

With the expression digital policy activism, we intend to refer to the production of both non-legislative (i.e., strategies, action plans, etc.) and legislative acts (i.e., already applicable regulations and directives, as well as proposals for such regulations or directives). A recent review of digital legislation identified as many as 50 of such EU legislative acts and proposals in the digital domain, of which 20 could be traced to the period 2019–2021 (Codagnone et al., 2021a, pp. 17–19). The number of non-legislative documents directly or indirectly related to the digital transitions and transformation released in the past 5 years amounts to 80 (Codagnone et al., 2021b). Both types of documents spiked since 2019 with the beginning of the new Commission chaired by Ursula von der Leyen, who launched a six priorities programme 2019–2024.^2^ Her priority ‘A Europe Fit for the Digital Age’^3^ figures as one of the most prominent ones and includes as horizontal pillars the Digital Strategy (Appendix Doc. 1) and the Digital Compass (Appendix Doc. 2) as well as 14 flagship initiatives.^4^ Since 2019 the Commission has been visibly busy and active in a wide variety of fields intervening through policy documents, legislative acts, or legislative proposals in AI, data spaces, online platforms, cybersecurity, industrial policy, technological industrial sectors (i.e., chips) and much more.

In the next section, we selectively review this policy bubble. Methodologically, we draw on document analysis following a systematic procedure based on Bowen (2009). According to Bowen (2009), researchers typically review previous literature as part of document analysis and incorporate the results of those reviews into their work. This step has been carried out in Section 2 of this study, which provides a review of previous literature on the key themes of our analysis, particularly digital sovereignty. The following part of our study is devoted to the analysis of primary sources, allowing us to use raw data as a basis for our analysis. This analysis is based on 30 institutional documents, including Commission communications, Commission staff working documents, and official legal texts that are reported in the appendix categorised by type and sector. The documents were selected based on two baseline inclusion criteria; their publication year (2019 or later) and their contextual relevance in digital policy. Specifically, we focus on five different policy domains: (1) the Digital Strategy and Compass; (2) the AI package; (3) the data package; (4) fairness and competition in digital markets; and (5) the industry package including the case of the Chips Act. We have selected these areas for the purpose of illustrating our claim. When elaborating on some of these documents specifically, we draw on the notation used in the Appendix (i.e., Doc. 1, Doc. 2, Doc. 20, etc.). It is worth mentioning that while document analysis has several advantages, such as, for instance, efficiency, availability, and transparency of documents or lack of obtrusiveness, it also exhibits a few limitations. One of those limitations refers to biased selectivity in the data collection process (Bowen, 2009). Therefore, there might be an unavoidable element of subjectivity in the selection of the 30 documents. The documents included in our analysis are used to verify findings and corroborate evidence against the background of previous literature and our conceptual framework (Angrosino & Mays de Pérez, 2000; Bowen, 2009). The systematic process of document analysis encompasses skimming (cursory review), reading (thorough review), and interpretation (Bowen, 2009). This iterative process “combines elements of content analysis and thematic analysis”, the latter being a form of ‘pattern recognition’ of emerging themes (ibid., p. 32), such as digital sovereignty and values in our case. In the following section, we provide a descriptive overview that emerged from the systematic process of document analysis, followed by a critical assessment in Section 4.2.

### Value Amplification in Europe’s Digital Policy: a Selective Review

*Digital Strategy and Compass*. The Digital Strategy for the new decade is presented in the Communication ‘Shaping Europe’s digital future’ (Appendix, Doc. 1) released in February 2020. It identifies four axes (technology for people; fair and competitive economy; open democratic and sustainable society; Europe as a global player), and contains a total of about 30 single priority actions. The Digital Strategy pays its rhetorical tribute to the mantra of sovereignty right at the outset when it declares that:

*“European technological sovereignty starts from ensuring the integrity and resilience of our data infrastructure, networks and communications. It requires creating the right conditions for Europe to develop and deploy its own key capacities, thereby reducing our dependency on other parts of the globe for the most crucial technologies. Europe’s ability to define its own rules and values in the digital age will be reinforced by such capacities. European technological sovereignty is not defined against anyone else, but by focusing on the needs of Europeans and of the European social model. The EU will remain open to anyone willing to play by European rules and meet European standards, regardless of where they are based*. *Citizens should be empowered to make better decisions based on insights gleaned from non-personal data. And that data should be available to all – whether public or private, big or small, start-up or giant. This will help society to get the most out of innovation and competition and ensure that everyone benefits from a digital dividend. This digital Europe should reflect the best of Europe - open, fair, diverse, democratic, and confident”* (Appendix, Doc. 1, p. 2).

It is worth stressing the emphasis on data on the one hand, and on the EU’s own rules and values (“open, fair, diverse, democratic and confident”) on the other, including the reference to the European social model. Equally interesting is the fact that the reference to technological sovereignty is immediately followed by a statement that emphasises fairness, competition, and open access to data. This can be seen as value amplification, where technical (infrastructure, networks, etc.) and organisational (capacities) issues are seen as instrumental to European values. Furthermore, technological sovereignty is presented as an instrument to protect the rights and needs of citizens and business and thereby linked to consumer protection and issues of competition. The unspoken targets are large technological corporations and online platforms that monopolise access to data. It is also worth considering how single actions are placed under some of the four axes. A white paper and future legislation on trustworthy and human-centric AI are the first priorities under the ‘technology for people’ axe, followed by various actions in different digital sectors. It should be noted that under the ‘fair and competitive economy’ the first key action is a European Data Strategy together with a package that addresses large platforms that act as *gatekeepers*. This implicitly means that in the data economy, markets are not yet contestable and competitive, which is thus clearly linked to the idea of sovereignty. This paves a clear way to what can be interpreted as the sovereignty-competition link existing in European digital policies and legislative proposals. Under the same axe, the Digital Strategy also lists the Industrial Strategy package that has the aim, again, to increase the competitiveness of European industries in key technologies. The package concerning platforms (Digital Services Act package) is then listed accordingly as a key measure under ‘open, democratic, and sustainable society’ in relation to disseminated contents and news. Under the ‘Europe as a global player’ axe, the discourse on sovereignty and global performance resurfaces with an appeal to Europe’s leadership in setting new rules and standards (i.e., GDPR and the Brussels Effect).

In March 2021, the Commission presented the Communication on “*Europe’s Digital Compass to a successful digital transformation of Europe by 2030*” (Appendix, Doc. 2) as a follow-up to the Digital Strategy. Under the heading ‘Joining Forces: Digital Transformation for Europe’s resilience’, the Digital Compass starts by stressing the opportunities and challenges raised by the Covid-19 pandemic. It calls on Europe to pursue empowering actions and to address weaknesses and vulnerabilities in order for Europe to attain digital sovereignty:

*“[Europe] needs to carefully assess and address any strategic weaknesses, vulnerabilities and high-risk dependencies which put at risk the attainment of its ambitions and will need to accelerate associated investments. That is the way for Europe to be digitally sovereign in an interconnected world by building and deploying technological capabilities in a way that empowers people and businesses to seize the potential of the digital transformation and helps build a healthier and greener society. In the State of the Union Address in September 2020, President von der Leyen announced that Europe should secure digital sovereignty with a common vision of the EU in 2030, based on clear goals and principles. The President put special emphasis on a European Cloud, leadership in ethical artificial intelligence, a secure digital identity for all, and vastly improved data, supercomputer, and connectivity infrastructures”* (Appendix, Doc. 2, p. 1).

Again, digital sovereignty is framed as a need for Europe to cope with its weaknesses, and as a justification to set ambitions high to ensure resilient and open strategic autonomy. In general terms, the Digital Strategy and the Digital Compass taken together seem to be based on a framing discourse emphasising the values of reaching certain targets or of undertaking certain actions. A concrete plan of objectives that matches the instruments to achieve them is missing. As such, they provide the foundation for the positive policy bubble that is substantiated and legitimised by the discourse of digital sovereignty.

*The AI package*. Since the first communication of 2018 (Appendix, Doc. 3), the Commission has issued a number of documents in the domain of AI (Appendix, Docs. 4–7), culminating with the proposal of the AI Act (Appendix, Doc. 4). AI is considered in fact a main pillar of the digital transformation both in the Digital Strategy and in the Digital Compass. In the AI package one finds a lot of text referring to human-centricity, ethics, and more broadly to the values of the European social model. This is a case of value amplification and policy framing informing the regulation of technology, which replicates to a large extent the discourses that preceded and followed the introduction of the GDPR. On the other hand, a careful look at both the AI Act proposal (Appendix, Doc. 4) and at its accompanying Impact Assessment (IA) (Appendix, Doc. 6), reveals the recurring theme of digital sovereignty. Specifically, the proposal contains the following statement:

“*A solid European regulatory framework for trustworthy AI will also ensure a level playing field and protect all people, while strengthening Europe’s competitiveness and industrial basis in AI. Only common action at Union level can also protect the Union’s digital sovereignty and leverage its tools and regulatory powers to shape global rules and standards*” (Doc. 4, p. 6).

This highlights how AI is related to the concern of creating a level playing field to protect digital sovereignty. It also implicitly reinforces the Brussels Effect (i.e., ‘shape global rules and standards’). In the accompanying IA, digital sovereignty features four times (Doc. 6, p. 13, p. 26, p. 32, p. 36) to stress the danger of fragmentation and the problem of a level playing field and to justify the need for intervention at EU level.

*The data package*. The European Strategy for Data (Appendix, Doc. 15), issued in February 2020, aims at establishing a path for the creation of European data spaces whereby more data becomes available for use in the economy and society. The objective of creating European data spaces is related to the discourse on digital sovereignty. It explicitly sets the goal of creating ‘*technological sovereignty in key enabling technologies and infrastructures for the data economy*’ (Doc. 15, p. 5). Further, it envisages that ‘*[t]he Commission will use its convening power as well as EU funding programmes to strengthen Europe’s technological sovereignty for the data-agile economy*’ (ibid., p. 16). In this context, the data-agile economy foresees fair access to data and the creation of a level playing field. For this purpose, the Data Strategy proposes an EU data framework that would support data sharing for innovators, particularly in the business-to-business (B2B) or government-to-citizens (G2C) domains. This should be enabled through open access to government data in sectors such as transportation and healthcare, as well as through privacy-preserving data marketplaces for companies to share data. The strategy aims to make the EU a pioneer in a data-driven society, with a Single Market where data flows freely across sectors, benefitting European businesses, researchers, and public administrations. To achieve such goals, actions with a regulatory component to set ‘clear and fair rules on access and reuse of data’ are foreseen. The two key legislative initiatives following up on the Data Strategy are the DA (Appendix, Doc. 18) and the DGA (Appendix, Doc. 16). In the accompanying IA of both acts (Docs. 17 and 19), digital sovereignty is mentioned both as an inspiring principle and as a final goal that the two acts would help achieve. In the IA of the DA (Doc. 19) concerns for EU data sovereignty in cloud and edge services are expressed. The document mentions the risk of unlawful access by non-EU or non-European Economic Area (EEA) governments to data stored in the cloud (Doc. 19, p. 14). The IA of the DGA stated that the act would “meet new market demands and allow the EU to become more competitive in the data-driven world economy, while maintaining its data sovereignty” (Doc. 17, p. 11). It is worth shedding light on how the DA proposal has been presented by two key EU policy-makers.

Margrethe Vestager, Executive Vice-President for a Europe Fit for the Digital Age, stated:

“*We want to give consumers and companies even more control over what can be done with their data, clarifying who can access data and on what terms. This is a key Digital Principle that will contribute to creating a solid and fair data-driven economy and guide the Digital transformation by 2030*”.^5^

Thierry Breton, Commissioner for Internal Market, commented that:

*“Today is an important step in unlocking a wealth of industrial data in Europe, benefiting businesses, consumers, public services and society as a whole. So far, only a small part of industrial data is used and the potential for growth and innovation is enormous. The Data Act will ensure that industrial data is shared, stored and processed in full respect of European rules. It will form the cornerstone of a strong, innovative and sovereign European digital economy”.*^6^

These two statements further support previously identified associations in the discourse on digital sovereignty, fairness, and competition.

*Fairness and competition: focus on online platforms.* The issues of fairness, competition, and level playing field have been directly addressed by a regulation already in place: The regulation for business users of online intermediation services, also known as the Platform-to-Business (P2B) Regulation (Appendix, Doc. 10) and two proposals for a new act on digital services and digital markets (DSA, Appendix, Doc. 11; DMA, Appendix, Doc. 12). Although their title and object are expressed in general terms, they clearly target online platforms. Platforms facilitate matching, reduce transaction costs, can foster innovation and help businesses in business intelligence, product development, and process optimization. On the other hand, their dominant position in the market indirectly fuels data-driven network effects (Recital 2 of P2B Regulation). They are the sources of data asymmetry which puts platforms in an advantageous position and makes users economically dependent on them. To address these issues, first the P2B Regulation was introduced in 2019, followed by the DSA and DMA proposals in 2020 and 2021 respectively.^7^ Together, the DSA and the DMA form the Digital Services Act package introduced by the Commission in December 2020. The Inception Impact Assessment, published four months earlier, clearly establishes the link between ensuring the contestability of digital markets and the EU’s digital sovereignty, arguing that “Europe’s estimated 10 000 online platforms are potentially hampered in scaling broadly and thereby contributing to the EU’s technological sovereignty, as they are increasingly faced with incontestable online platform ecosystems” (European Commission, 2020, p. 2). This essentially reinforces the assumption that Europe can achieve technological sovereignty by strengthening antitrust mechanisms for gatekeepers. It also aligns with the recurring geopolitical narrative that Europe’s own digital sovereignty depends on the performance and activities of non-European digital players. This again demonstrates the tendency of European policy-makers to exercise regulatory power via the Brussels Effect.

*The industry package and the special case of the Chips Act*. The Industrial Strategy of 2020 (Appendix, Doc. 22), its update of 2021 (Appendix, Doc. 24), and the SME (small and medium-sized enterprises) Strategy for Sustainable and Digital Europe (Appendix, Doc. 23) all seem to be committed to industrial and technological sovereignty. The Industrial Strategy contains several references to it: “The need for Europe to affirm its voice, uphold its values and fight for a level playing field is more important than ever. This is about Europe’s sovereignty” (Doc. 22, p. 1). “With its Strategy on Shaping Europe’s Digital Future, the Commission set out its vision for how Europe can retain its technological and digital sovereignty and be the global digital leader” (ibid., p. 4). “The EU also needs to ensure that its Intellectual Property policy helps to uphold and strengthen Europe’s tech sovereignty and promote global level playing field” (ibid., p. 5). “Europe’s digital transformation, security and future technological sovereignty depends on our strategic digital infrastructures.” (ibid., p. 13). The link between sovereignty and the frequent association with the level playing field is hard to miss, highlighting once more the role of framing and value amplification. The SME Strategy also makes several references to sovereignty (Doc. 23, pp. 2, 10, and 15), stating that the 25 million European SMEs are central to economic and technological sovereignty. Their need to find capital abroad to expand poses a risk to Europe’s technological sovereignty, growth, and jobs. On the other hand, the 2021 update of the Industrial Strategy shifts its focus from digital sovereignty to the related ‘Open Strategy Autonomy’, a new frame illustrated in the Trade Policy Review (Appendix, Doc. 26). These three strategic documents contain references to many initiatives and actions without a clear indication of the underlying funding mechanism. This suggests a certain degree of voluntarism. The SME Strategy places a strong emphasis on fully unlocking the potential of small companies by reducing regulations and administrative burden, which, as we will demonstrate in Section 4.2, stands in contrast to the introduction of new regulations. The updated Industrial Strategy is accompanied by a working document reviewing strategic dependencies and vulnerabilities that contains a lengthy section on semiconductors (Appendix, Doc. 25, pp. 82–90). In this section, the document provides a detailed analysis of the chips industry at global level, its supply chain, and, in particular, Europe’s weaknesses compared to its global competitors (China, South Korea, Taiwan and the USA). In light of this analysis, the Commission unveiled its proposal for a Chips Act in February 2022 (Appendix, see both Docs. 20 and 21). The title of the press release announcing the act is telling: “Digital sovereignty: Commission proposes Chips Act to confront semiconductor shortages and strengthen Europe's technological leadership”.^8^ In a single title, we find both digital sovereignty and technological leadership, which indicates that value framing is at least as important as the underlying policy goal.

### The More, the Better? A Critical Assessment

*Digital Strategy and Compass*. The Digital Strategy, as mentioned earlier, encompasses more than 30 actions, some of which are policy documents (i.e., white paper), others legislative acts (i.e., the Digital Service Act packages), and still others technological investments. On the other hand, on the indication of the exact amount of source of funding, the Digital Strategy remains at a generic level. The document also discloses little information about the interplay and interactions among the 30 different actions and about a coherent and consistent vision more generally. The Digital Compass lists 10 ambitious targets, of which we selectively discuss a few in the following. The target for semiconductors aims at doubling the EU share in their global production by 2030, that is, from 10 to 20%. This seems ambitious given the current relative position of Europe compared to its global competitors. We will revisit this argument when discussing the industrial package and the related Chip Act (Appendix, Docs. 20 and 21). The cloud market is currently dominated by non-EU hyperscalers. Consequently, the Compass sets the objective of establishing “10 000 climate neutral highly secure edge nodes [by 2030, which] are deployed in the EU [and] distributed in a way that will guarantee access to data services with low latency (few milliseconds) wherever businesses are located” (Appendix, Doc. 2, p. 2 of the Annex). The starting baseline is zero, indicating that this is an open market with no incumbents, where Europe could possibly enhance its competitiveness by 2030. The Gaia-X project is intended to underpin this objective, but it will require funding mechanisms that reflect the same level of ambition. Targets that appear more challenging to reach instead are those concerning SMEs’ use of AI and big data, and of reaching in 90% of the cases a basic level of digital intensity. Taking into account the 2020 baseline, these targets can hardly be met without an exogenous jolt of investments by 2030. The same applies to the target of doubling the number of European unicorns (from 107 to 214) by 2030, which would require new funding mechanisms. A concrete strategic analysis of Europe’s strengths and weaknesses should accompany this target and make it less generic. Overall, it seems that the Digital Compass lacks a map with clear instructions and a general strategic vision of the policy interdependencies and how they might contribute to reach the declared targets (Codagnone et al., 2021b, pp. 43–47). There is no clear indication of the funding sources to reach such targets.

*The AI package.* The policy discourse developed around AI has three dimensions: to support the technological and industrial capacity of the EU and the adoption of AI, prepare for socio-economic changes, and ensure an appropriate ethical and legal framework. The Commission has established a High-Level Expert Group on AI representing a wide range of stakeholders and has tasked it with drafting AI ethics guidelines and preparing a set of recommendations for broader AI policy. The Group drafted AI Ethical Guidelines,^9^ which, among others, identify seven key requirements that AI applications should respect to be considered trustworthy.^10^ This work informed the White Paper on AI – A European Approach to Excellence and Trust (Appendix, Doc. 5). The white paper strongly emphasises the need for a human-centric, transparent, and ethical AI. In parallel, the AI Act proposal was developed. The Act divides AI systems into three categories: unacceptable-risk AI systems, high- risk AI systems, and limited- to minimal-risk AI systems. High-risk systems would be subject to the largest set of requirements, including human oversight, transparency, cybersecurity, risk management, data quality, monitoring, and reporting obligations. Enforcement could include fines of up to €30 million or six percent of global revenue, making penalties even heftier than those incurred by violations of GDPR. The Act set the ambitious goal of bringing all AI initiatives under the supervision of one single authority to monitor all possible AI systems and uses. It must be noted that given the horizontal character of AI systems, they have clear interplay and dependencies with other technological domains. This corresponds to the AI Act with other related policy and legal acts. These legal acts concern, among others, cybersecurity, data governance, infrastructure, digital services, digital markets, and liability (Codagnone et al., 2021a).

*The data package*. As part of the data package, the DGA is presented as a way to increase trust in data sharing, strengthen mechanisms to increase data availability, and overcome technical barriers to data reuse. The regulation aims at supporting common European data spaces in strategic domains, involving both private and public players in various sectors (i.e., health, environment, energy, agriculture, mobility, finance, manufacturing, public administration and skills). It builds on three pillars: to enable greater data sharing among public and private sector entities; to establish a notification and compliance framework for providers of data sharing services with the aim of creating more trustworthy data sharing; and to establish a (voluntary) registration regime for data-altruist entities. It also sets out a legal framework for the reuse of public sector data that are covered by third-party rights. Further, the DGA includes rules regulating international transfers of non-personal data by a reuser that was granted access to such data by the public sector. A sort of replication of some of the features of the GDPR has been noted in the DGA, visible in the definition of new actors and in the institution-building provisions (Papakonstantinou & De Hert, 2021). New terms are introduced in Article 2 of the DGA: ‘data holders’, ‘data users’, ‘data’, or ‘data sharing’ (Article 2). They are the counterparts of GDPR’s ‘data subjects’, ‘controllers’, ‘personal data’ and ‘processing’ (in Article 4). The DGA would establish a new authority to monitor all of the above (Articles 12, 13 and Chapter V) and for cooperation, suggests a European Data Innovation Board (Article 26), whose name reminds of the GDPR’s European Data Protection Board, an administrative body endowed with legally binding powers. According to the press release presenting the proposal for the DA in February 2022,^11^ the DGA would regulate the processes and structures to facilitate data sharing by companies, whereas the DA would regulate those who can create value from data under certain conditions. The measures of the proposed DA encompass: the provision of access to users of connected devices to data generated by them; the protection of SMEs to avoid that they are contractually abused in data sharing agreements; and facilitating access for public sector bodies to use data held by the private sector that is necessary for exceptional circumstances.

*Fairness and competition: focus on online platforms.* The P2B Regulation addresses the relationship between platforms and businesses and introduces fair rules for a predictable business environment for smaller businesses and merchants on online platforms. The regulation argues that that the gateway position of online platforms increases the risk of harmful trading practices. This line of argumentation is brought further with the DSA and the DMA proposals, which the Commission considers centre pieces of the Digital Strategy. The DSA aims at protecting consumers and their fundamental rights, establishing a transparent and accountable framework for online platforms. The DMA sets some criteria for qualifying a large online platform as a gatekeeper and aims to ensure that such gatekeepers behave in a fair way online. Specifically, it contains provisions that impose obligations on gatekeepers to share data with business users (Article 6). In the case of personal data, the sharing and processing mechanisms are subject to GDPR. For data providers and data recipients, both the obligation to share and the right to receive data in a continuous and real-time manner regulated under GDPR may be a regulatory and technical challenge. Moreover, it is worth noting that on the other side of the Atlantic, both files received criticism for representing excessive ex ante precautionary approaches to antitrust imposed on predominantly US tech companies (Broadbent, 2020; Portuese, 2021).

*The industry package and the special case of the Chips Act*. The target of doubling the share of production of chips by 2030 announced in the Digital Compass seems ambitious. Nevertheless, as anticipated in the fall of 2021, in February 2022 the Commission unveiled its proposal for a Chips Act (Appendix, see both Docs. 20 and 21). The Act has a clear aim to reinforce the role of the EU in shaping the global value chain of semiconductors. Using a slogan, the objective of the Chips Act can be summed up as moving from the lab to the fab, from R&D to production. It has three pillars. First, traditional investments in R&D to be funded by Horizon Europe, Digital Europe, and the Key Digital Technologies Joint Undertaking under the umbrella of a new Chips for Europe Initiative. Second, a new state aid exemption for cutting-edge foundries (semiconductor manufacturing plants). With this pillar, the Commission wants to increase capacity in the most concentrated and capital-intensive stage of chip production: fabrication. The Chips Act allegedly will allow EU countries to grant subsidies for manufacturers willing to build cutting-edge ‘mega-fabs’ in the EU. Given the global level of subsidies already in place by EU competitors around foundries, this seems to be a very eager and expensive goal. The third pillar is the introduction of measures to monitor the supply chain and intervene during crises. Despite the plans laid out in the Chips Act, the mobilization of funds to realise this piece of legislation remains unclear and will depend on how much private investment can be attracted to supplement the public funds. According to the Commission, €43 billion of public and private investment will be mobilised, of which €11 billion will come from the The Chips for Europe Initiative. From these €11 billion, the EU itself will provide only €3 billion through the redirection of other funds. So, for the remaining funds, the Act merely provides the framework that countries, private firms, and investors can use. Public and private funds will have to come mostly through the instrument of the Important Project of Common European Interest (IPCEI) on microelectronics. Compared to the USA, venture capital markets in Europe are proving to be insufficiently developed enough to support such large technological investment projects.

## Discussion

### Implications of Positive Policy Bubbles

All of the documents (both non-legislative initiatives, legislative acts, as well as legal proposals) reviewed so far and reported in the appendix can be critically appraised on the account that supports our claim that the oversupply of digital policies does not necessarily produce a coherent and clear picture. Regulatory oversupply emerging from a positive policy bubble leads to a lack of a general vision and increases the gap between reality and political-economic ambitions, especially in terms of the required investments. The reviewed policies and legislative proposals, as well as the plans of the Digital Strategy and Digital Compass, lack an overall coherent strategic vision that clarifies the synergies between the policy packages to avoid inconsistencies between objectives and targets. Digital policies and legislation are not fully harmonised, particularly the competition, industrial, and consumer protection packages. There is no concrete integration between the objectives of protecting citizens and taming big businesses with an industrial policy that will boost European industry and help create large and new European companies. A good and integrated policy framework would be one where non-legislative initiatives, legislative acts, funding mechanisms, and alliances are combined and balanced in a policy approach that represents multiple interests. This is not the case for the policies and legislative proposals reviewed so far. One such contradictions can be exemplified by the SME strategy’s objective to reduce administrative burden, whereas some of the proposed legislative acts will increase compliance efforts, adding to those already imposed by the GDPR. This is particularly true for the AI Act, which, as the GDPR, will create more obstacles for innovative SMEs than for large incumbents. It conveys the impression that there is an excessive reliance on regulation without a thorough appraisal of the costs imposed on businesses to deal with administrative burden, conformity tests, and audits. The proposal for the AI Act, if adopted, would be challenging for most companies, especially for smaller software providers. With the GDPR, the most influential data protection legislation worldwide, Europe has stood out as a regulatory champion, without fully considering the challenges that such regulation places on European AI SMEs. It seems as if the EU is focussing on regulation to make up for the gaps and weaknesses of its positioning in the digital data ecosystem. We have shown that frequently the discourse places sovereignty in the context of leveling the playing field to justify regulatory interventions to take back control, as is the case with the DSA and DMA. In addition, the funding mechanisms and the funds available seem inadequate, and mobilisation is sometimes unclear given existing gaps (Codagnone et al., 2021b, pp. 41–43 and 59–60). The ambitions do not appear to be aligned with the reality and risk to be overly voluntaristic. Given limited financial resources, digital sovereignty and digital autonomy require strategic political choices. However, such strategic choices are not made as the planned initiatives are spread over all possible domains. The Chips Act is a special case in the gap between ambition and reality. The European Chips ambitions have been criticised in terms of feasibility and implementation with regard to the current relative positions of Europe and its global competitors, the capital-intensive nature of building foundries, and the required specialization in this industry (Kleinhans & Baisakova, 2020; Hancké & Garcia Calvo, 2022; Poitiers & Weil, 2022). Investing in the manufacturing of mature semiconductors seems to be a good idea for Europe only to a limited extent. Instead, the EU should focus on its strengths, where it can leverage its skilled workforce and excellent world-class network of research laboratories. Rather than facing a comparative disadvantage, it could have a comparative advantage in mature chip manufacturing. Considering the costs of setting up and operating chips fabrication plants, which the strategic dependencies staff working document has laid out in detail (Doc. 25, p. 82), the funds directly mobilised already in the Chips Act seem insufficient, and it remains unclear if the additional funds needed will be provided by public and private investors.

### Act-itifcation and Legal Incoherence

Our analysis led to two additional observations that were not covered by our conceptual framework. First, we noticed that in less than 2 years, the Commission has presented a tsnumai of legislative initiatives taking the shape of a total of six ‘Acts’, including the AI Act, the DGA, the DA, the DSA, the DMA, and the Chips Act. This suggests that since the introduction of the GDPR, the preferred tool to regulate digital is now regulations rather than directives (Papakonstantinou & De Hert, 2022; Papakonstantinou & De Hert, 2021). Regulations are directly applicable and do not need the localised transposition through national legislation. Moreover, a very recent paper observed a notable characteristic of this new wave of proposals for legislative acts, termed *act-ification* (Papakonstantinou & De Hert, 2022).^12^ The authors noticed the novelty of naming the legislative proposal (i.e., AI Act, Data Act, etc.), rather than using the more traditional anonymus, indicating only the number followed by the full general title (i.e., Regulation (EU) 2019/1150, etc.). According to Papakonstantinou and De Hert (2022), this choice is motivated by the goal of having such acts to be immediately recognisable by name. This is due to their ambition to regulate wide and important spaces of everyday life, instead of being directed to a restricted set of technical stakeholders, a dynamic that can also be observed in the proposed framework for a European Digital Identity (Weigl et al., 2022). Once the act-ification process is accomplished, one could envisage a new European domain for the regulation of the digital domain. Although this naming practice appears only to be a formal and nominal element, together with the sheer volume of legislative acts, these trends could be interpreted as a manifestation of the EU’s digital constitutionalism. Thereby, entire swathes of activities are regulated from scratch and, to some extent, without considering existing national legislation. According to Pagallo (2022), also the notion of digital sovereignty “echoes mechanisms of centralization” and tends to neglect the consideration of alternative modes of collaboration.

The second observation, a potential consequence of policy oversupply and act-ificiation, is about the complexity, inconsistency, and lack of coherence that the new proposals for legislative acts are poised to produce. This has been shown in the most recent and thorough critical assessment of digital legislation contained in a report delivered to the European Parliament (Codagnone et al., 2021a, pp. 53–72). This report shows that the interplay and interdependency of the AI Act with other pieces of legislation (on cybersecurity, privacy, liability, and in relation to both the DSA and DMA) can generate problems of regulatory coherence and uncertainty. The same also applies to the interplay and dependencies between the P2B regulation and the DMA, and between the DSA and the e-Commerce Directive.

## Conclusion

In this paper, we argued and showed that in the past few years, EU action in the digital domain has been characterised by policy and regulatory activism. We consider this a case of a positive policy bubble with an oversupply of policies and legislative proposals. Our argument is that policy framing in terms of digital sovereignty, followed by digital constitutionalism, has influenced such activism beyond concrete policy instrumentalism. This has resulted in an increasing complexity of policies and regulations that lack coherence and consistency.

While preserving fundamental rights, protecting consumers and businesses, ensuring contestable markets, and an open, democratic society are among the declared goals of much of the reviewed policy documents and legislative proposals, there is also the side effect of overregulation and mismatch between declared objectives and reality. The ambition to strengthen digital and technological sovereignty in all domains, even when this seems unrealistic (i.e., the case of Chips) or when there is nothing upon which to take back control (i.e., because Europe never had such control, as in the case of online platforms), appears as a new form of policy voluntarism with a *techno-Gaullism* bent.

Digital sovereignty and digital autonomy do not come for free and require strategic policy decisions on the allocation of scarce resources across a wide range of actions. Choices that we did not find clear and unambiguous in our review of policies and legislative proposals. It seems as if the Brussels Effect euphoria has convinced EU policy-makers that sovereignty and autonomy can be gained by regulating others, which is questionable. Autonomy and sovereignty require the building of capacities and innovation in the various digital domains. The AI Act, for instance, will regulate high-risk systems and protect individuals, but in itself does not ensure that European AI firms will become more innovative and will increase in number so that Europe will achieve a dominant position. It is highly possible that the newly proposed acts protect fundamental rights and uphold European values and principles in the digital domain, but it is unlikely that they will create the needed capacities and innovation. It remains doubtful that the DSA and DMA will place Europe in a dominant position with respect to foreign tech giants to create new sources of sovereignty and autonomy for Europe in the data economy. The Data Strategy together with the DGA and DA have the goal of creating new data spaces where European citizens and businesses are in the driving seat. This requires mora data exchanges between businesses (B2B), from businesses to public authority (B2G) or vice versa (G2B). Yet, such exchanges will not simply occur because of new regulation, as they depend on the structure of incentives and on clear business models that are yet to emerge. The side effects of digital policy activism at the EU level include the risk of increasing the administrative burden, especially for SMEs, and stifling innovation, as well as creating more uncertainty due to regulatory inconsistency and lack of coherence.

In conclusion, the answer to the question included in the title of our paper ‘the more, the better?’ remains highly debatable. Certainly, there is also a comforting prospect in the EU’s approach to regulating the digital. The current set of proposals is commendable as they provide a stable basis to protect consumers and create a level playing field for European businesses. Drawing on our analysis and findings, we argue that policy and regulatory parsimony, however, carries its value and there might be a silver lining in striving for better integration between the different pieces adding up to the bloc’s digital policy. Rather than a response to the powerful position of foreign private enterprises, digital sovereignty should be a means, not an end, in EU policy-making to attract investments, foster innovation, complete the Digital Single Market, and adopt harmonised standards.

## Appendix: Documents Reviewed

### General Strategic Communications

1. European Commission. (2020). Shaping Europe’s Digital Future. COM(2020) 67 final, Brussels.
2. European Commission. (2021). 2030 Digital Compass: The European Way for the Digital Decade. COM(2021) 118 final, Brussels.

### Artificial Intelligence Package

3. European Commission. (2018). Artificial Intelligence for Europe. COM(2018) 237 final, Brussels.
4. European Commission. (2021). Proposal for a Regulation of the European Parliament and of the Council on Laying Down Harmonised Rules on Artificial Intelligence (Artificial Intelligence Act) and Amending Certain Union Legislative Acts. COM(2021) 206 final, Brussels.
5. European Commission. (2020). White Paper. On Artificial Intelligence—A European Approach to Excellence and Trust. COM (2020) 65 final, Brussels.
6. European Commission. (2021). Commission Staff Working Document. Impact Assessment accompanying the Artificial Intelligence Act. SWD (2021) 84 final, Brussels.
7. European Commission. (2021). Fostering a European Approach to Artificial Intelligence. COM(2021) 205 final, Brussels.

### Cybersecurity Package

8. Regulation (EU) 2019/881 of the European Parliament and of the Council of 17 April 2019 on ENISA (the European Union Agency for Cybersecurity) and on Information and Communications Technology Cybersecurity Certification and Repealing Regulation (EU) No 526/2013 (Cybersecurity Act).
9. European Commission. (2020). The EU's Cybersecurity Strategy for the Digital Decade. COM(2020) 823 final (also known as the NIS2 directive).

### Digital Services Package

10. Regulation (EU) 2019/1150 of the European Parliament and of the Council of 20 June 2019 on promoting fairness and transparency for business users of online intermediation services (also known as P2P regulation).
11. European Commission. (2020). Proposal for a Regulation of The European Parliament and of The Council on a Single Market for Digital Services (Digital Services Act) and amending Directive 2000/31/EC. COM(2020) 825 final, Brussels.
12. European Commission. (2020). Proposal for a Regulation of The European Parliament and of The Council on Contestable and Fair Markets in the Digital Sector (Digital Markets Act). COM(2021) 206 final, Brussels.
13. European Commission. (2020). Commission Staff Working Document. Impact Assessment accompanying the Digital Services Act. SWD(2020) 348 final, Brussels.
14. European Commission. (2021). Commission Staff Working Document. Impact Assessment accompanying the Digital Markets Act. SWD(2020) 363 final, Brussels.

### Data Package

15. European Commission. (2020). A European Strategy to Data. COM(2020) 66 final, Brussels.
16. European Commission. (2020). Proposal for a Regulation of the European Parliament and of the Council on European data governance (Data Governance Act). COM(2020) 767 final, Brussels.
17. European Commission. (2021). Commission Staff Working Document. Impact Assessment accompanying the Data Governance Act. SWD(2020) 295 final, Brussels.
18. European Commission. (2022) Proposal for a Regulation of the European Parliament and of the Council on harmonised rules on fair access to and use of data (Data Act), COM(2022) 68 final, Brussels.
19. European Commission. (2021). Commission Staff Working Document. Impact Assessment accompanying the Data Act. SWD(2020) 34 final, Brussels.

### Chips Act

20. European Commission. (2022). A Chips Act for Europe. COM(2022) 45 final, Brussels.
21. European Commission. (2022). Proposal for a Regulation of The European Parliament and of The Council on establishing a framework of measures for strengthening Europe's semiconductor ecosystem (Chips Act). COM (2022) 46 final, Brussels

### Industry Package

22. European Commission. (2020). A New Industrial Strategy for Europe. COM(2020) 102 final, Brussels.
23. European Commission. (2020). An SME Strategy for a sustainable and digital Europe. COM(2020) 103 final, Brussels.
24. European Commission. (2021). Updating the 2020 New Industrial Strategy: Building a stronger Single Market for Europe’s recovery. COM(2021) 350 final, Brussels.
25. European Commission. (2021). Commission Staff Working Document. Strategic dependencies and capacities. SWD(2021) 352 final, Brussels.
26. European Commission. (2021). Trade Policy Review—An Open, Sustainable and Assertive Trade Policy. COM(2021) 66 final, Brussels.

### Funding

27. European Commission. (2020). Europe's moment: Repair and Prepare for the Next Generation. COM(2020) 456 final, Brussels.
28. European Commission. (2020). Commission Staff Working Document. Identifying Europe's recovery needs. SWD(2020) 98 final, Brussels.
29. European Commission. (2021). Recovery and Resilience Plans. Example of component of reforms and investments –Digital connectivity (retrieved from: https://ec.europa.eu/info/sites/default/files/component_digital_connectivity.pdf, 10 July 2022).
30. European Commission. (2021). The EU’s 2021–2027 long-term Budget and NextGenerationEU. Facts and Figures. Luxembourg: Publications Office of the European Union.

## Footnotes

1 Hereinafter referred to as Commission.

2 See: https://ec.europa.eu/info/strategy/priorities-2019-2024_en

3 See: https://ec.europa.eu/info/strategy/priorities-2019-2024/europe-fit-digital-age_en

4 Flagship initiatives listed in no particular order: AI, Data Strategy, Industrial Strategy, Chips Act, DMA, DSA, Digital Identity, High Performing Computing, Digital Skills, Cybersecurity, Space, Connectivity, Contributing to European Defence, EU-US Trade and Technology Council.

5 *Ibid*.

6 *Ibid*.

7 The DMA entered into force on 1 November 2022, and the DSA entered into force on 16 November 2022.

8 European Commission, Press Release of 8 February 2022 ‘Digital sovereignty: Commission proposes Chips Act to confront semiconductor shortages and strengthen Europe’s technological leadership’ (https://ec.europa.eu/commission/presscorner/detail/en/ip_22_729).

9 Ethics Guidelines for Trustworthy AI, 8 April 2019 (https://ec.europa.eu/digital-single-market/en/news/ethics-guidelines-trustworthy-ai).

10 The seven key requirements are human agency and oversight; technical robustness and safety; privacy and data governance; transparency; diversity; non-discrimination and fairness; societal and environmental well-being; accountability.

11 European Commission, Press Release of 23 February 2022 ‘Data Act: Commission proposes measures for a fair and innovative data economy’ (https://ec.europa.eu/commission/presscorner/detail/en/ip_22_1113).

12 Papakonstantinou and De Hert (2022) also discuss a second and a third trend they call ‘GDPR mimesis’ and ‘regulatory brutality’ which go, however, beyond the scope of this paper.
